# Fatigue Performance of the CA Mortar Used in CRTS I Ballastless Slab Track under Simulated Servicing Condition

**DOI:** 10.3390/ma11112259

**Published:** 2018-11-13

**Authors:** Yuchuan Shan, Shuguang Zheng, Xuefeng Zhang, Wei Luo, Jingda Mao, Deyu Kong

**Affiliations:** 1 Zhejiang University of Technology Engineering Design Group Co., Ltd., Hangzhou 310014, China; adrisyc@zjut.edu.cn (Y.S.); zc05@163.com (S.Z.); zhangxue_feng@126.com (X.Z.); 2 College of Architecture & Civil Engineering, Zhejiang University of Technology, Hangzhou 310023, China; lw@shzf.com.cn (W.L.); mjd1987517@163.com (J.M.); 3 Shanghai Architectural Design Co., Ltd., Shanghai 200021, China; 4 Zhejiang Provincial Institute of Communications Planning, Design & Research, Hangzhou 310006, China

**Keywords:** ballastless slab track, cement asphalt mortar, fatigue, mechanical characteristic, high-frequency vibration

## Abstract

The cement and asphalt mortar (CA mortar) used in the China Railway Track System (CRTS) I ballastless slab track may encounter a coupling fatigue effect under the high-frequency vibration, load and high-and-low temperature cycles, and the deterioration under fatigue may happen during service of the high-speed railway. In this study, the performance degradation and its mechanism of the CA mortar with and without polymer emulsion incorporated under the coupling fatigue effects of the high-frequency vibration, load and temperature were studied by using an anti-fatigue testing device specially developed for the CA mortar used in the ballastless slab track of the high-speed railway. The results showed that the deformation capacity of the CA mortar for CRTS I slab ballastless slab track decreased after fatigue test under simulated service environment, presenting a typical brittle characteristic and an obvious reduction of the ductility and toughness. The Scanning Electron Microscopy (SEM) observation and the mercury intrusion porosimetry (MIP) analysis showed that the volume of the macropore decreased whereas that of the micropore increased after the fatigue test. The asphalt in the hardened CA mortar revealed a softening and migration from the bulk paste to fill the pore and make the structure denser and even ooze out of the CA mortar under the high-frequency vibration and high temperature. Through incorporating the polymer emulsion, the anti-fatigue property of the CA mortar was obviously improved, which can prevent the CA mortar from losing its elastic adjustment function too early. Though increase of the strength and elastic modulus for the CA mortar after severe service is beneficial to the stability of train running, the comfort level and safety of the train operation may decline due to the gradual reduction of the ductility & toughness and the gradual loss of the elastic damping adjustment function of the CA mortar between the base concrete slab and the track slab.

## 1. Introduction

Because of its manifold advantages, including the reduction in structure height, the lower maintenance requirements and the increased service life, the ballastless slab track has been widely used in the construction of high-speed railway (HSR) in many countries, such as Japan [1], Germany [2] and now in China [3]. In the structure of the ballastless slab track, the cement asphalt mortar (hereinafter abbreviated CA mortar) injected between the concrete roadbed and the track plate is one of the most important parts, which functions as a cushion layer to play important roles such as supporting, adjusting, load transferring, vibration absorption and shock insulation, etc., and improve the durability of the track structure as well as the ride safety and comfort level of the high-speed trains [1,2,3].

The CA mortar is an organic-inorganic composite with mechanical properties that are highly sensitive to time and temperature, consisting of cement, asphalt and/or polymer emulsion, fine aggregate and chemical admixtures, such as superplastisizer, expansive, air-entrained, and de-foaming agent, etc. Two kinds of CA mortar are often used according to the structure of the ballastless track, one with high elastic modulus (7000–10,000 MPa) and high compressive strength (more than 15.0 MPa at the curing age of 28 days is required) used in Bögl slab track firstly developed in Germany [2], in which anionic asphalt emulsion is utilized, and another one with low elastic modulus (100–300 MPa) and low compressive strength (only require a compressive strength of over 1.8 MPa at 28 days) previously employed in the shinkansen slab track [1], which uses cationic asphalt emulsion as the main raw material. Both CA mortars are now introduced into China and employed in the construction of high-speed railway, i.e., China Rail Track System, CRTS I and CRTS II. The CRTS I ballastless slab track adopts CA mortar with low elastic modulus, whereas CRTS II ballastless slab track employs CA mortar with high elastic modulus [3].

In recent ten years, a large HSR network has been built in China and more than 6000 km of HSR lines implemented with CRTS I or II ballastless slab tracks. Numerous studies, especially in China, have been or are being carried out and reported on fabrication [4] and improved properties [5], the rheological behavior of the fresh CA mortar [6,7,8,9], the mechanical properties [10,11,12,13,14,15], the dynamic or impact characteristics [16,17,18,19], the creep [20], and the durability of the hardened CA mortar. For example, the mixing kinetics of CA mortar was studied by using a power consumption method and the theory of air entrainment was used to interpret the effects of mixing speed, fluidity and mixing time on the air content of the mortar [4]. In order to improve properties of the hardened CA mortar, a novel mixing method was developed and the colloidal silica sol was incorporated to improve the expanding stability and to avoid bleeding of the fresh CA mortar during hardening [5]. About the rheological behavior of the fresh cement asphalt paste and mortar, influence of many factors, such as type of asphalt emulsion, superplasticizer dosage, water to cement ratio, viscosity-modifying agent, solid volume fraction (Vs), mass ratio of asphalt to cement (A/C), have been investigated and discussed in details [6,7,8]. A predictive model of CA composite particles was also proposed to determine the maximum particle packing density of CA pastes by the maximum particle packing density of cement paste, the maximum particle packing density of asphalt emulsion, and the volume fraction of asphalt in asphalt-cement system [9].

After hardening, it has been revealed that the framework formed by the hardened cement paste was the primary skeleton and the asphalt phase a filling phase for the CA with high elastic modulus, but for the CA mortar with low elastic modulus, the demulsified asphalt exhibited as a dominant phase, thus two weak structure skeletons, formed by the asphalt membrane and the hardened cement paste, respectively, were responsible for the strength [10]. Because of the existence of the asphalt phase, the compressive and dynamic behavior of the CA mortar were both found to be highly dependent on the temperature and the loading rate [11,12,13,14,15,16,17,18,19]. Besides, the creep of the CA mortar on different load levels were also studied by using a developed creep testing apparatus and for the CA mortar, two creep stages, i.e., the attenuation creep stage in which the deformation rate decreases gradually and the steady creep stage in which the creep rate is relatively stable were found. A creep model based on the thermodynamic theory was then established to help improve the structural design theory of ballastless slab track [20].

For the engineering structures, the durability has been paid much attention in the worldwide. For the CA mortar, the most noteworthy is its anti-fatigue performance under the action of high frequency vibration. Studies have shown that [21] the high-speed train will induce a strong high-frequency vibration on the track structure caused by the ground waves when the train speed reaches or exceeds a certain critical speed, which will affect the safety and comfort of the high-speed train. In serious cases it may cause the train derailment. The natural frequencies of the CA mortar are significantly different from those of track plate and concrete base, which plays an important role in preventing resonance in high-speed train operation [22]. Therefore, the fatigue degradation of the CA mortar by high frequency vibration should not be ignored. According to a practical investigation on the test section of Qin-shen high-speed railway [23], although the conventional performance and anti-freeze performance of the CA mortar meet the requirements, there still existed some durable problems. After 1-2 years of actual operation, the elastic modulus of the CA mortar used increased significantly and the local fragmentation occurred.

According to Xiang Jun et al. [24], the fatigue deterioration of the CA mortar not only reduces its own function of vibration and noise reduction, but also significantly affects the dynamic performance of the ballastless slab track. Compared with the normal working state of the CA mortar, the deterioration of the CA mortar will cause the track slab to be suspended, which will cause the vertical acceleration of the track slab to increase more than 10 times and the displacement to increase more than 20 times. The pressure of the rail on the slab increases much sharply. As the running speed increases, other dynamic response values of the system also increase rapidly. Therefore, in the maintenance of the ballastless slab track, the deterioration of the CA mortar should be carefully avoided.

However, there is no anti-fatigue performance requirement for the CA mortar used in CRTS I ballastless slab track. For the CA mortar used in CRTS II ballastless slab track, although an anti-fatigue performance is required, its frequency of the dynamic load is only 5 Hz in the testing method. Wang et al. [25] studied the fatigue characteristics of the CA mortar under various ambient temperatures, stress levels and sine wave loading conditions, but the loading frequency was only 10 Hz. However, with the increase of the running speed of the train, a very strong high-frequency vibration on various structural layers of the slab track may be produced and the frequency can reach as high as up to more than 50 Hz [26], and that is why a cushion layer such as the CA mortar is used to insulate the high-frequency vibration to improve the degree of comfort while taking the high-speed train. Typically, the CA mortar as a vibration-insulating layer will encounter this high-frequency vibration and the deterioration under fatigue may happen during service of the high-speed railway. In this study, the performance degradation and its mechanism of the CA mortar with and without polymer emulsion incorporated under the coupling fatigue effects of the high-frequency vibration, load and temperature were studied by using an anti-fatigue testing device specially developed for the CA mortar used in the ballastless slab track of the high-speed railway [27].

## 2. Experimental

### 2.1. Materials

The CA mortar used in the study was prepared by using a slow-crack rapid-coagulation cationic asphalt emulsion and a dry-mixed cement mortar, both of which were specially designed and produced for the CA mortar used in CRTS I ballastless slab track of the high-speed railway, provided by Hubei Guochuang High-tech Group Co., Ltd. (Wuhan, China) and Wuhan Aojie New Building Materials Co., Ltd. (Wuhan, China), respectively. The properties of the bituminous emulsion and the dry-mixed cement mortar are listed in Table 1 and Table 2, respectively. While preparing the CA mortar, a polymer emulsion with a solid content of 47.2% (mass fraction, same as below) provided by Wuhan University of Technology (Wuhan, China), was also used in the study. An HG-PCA600 polycarboxylic acid superplastisizer with a solid content of 35% and a water-reducing rate of about 28% produced by the admixture factory of Hangzhou Building Components Group (Hangzhou, China), was used while preparing the CA mortar. The de-foaming agent used in this study was an organic silicon de-foaming agent provided by Zhejiang Lanting High-tech Group Co., Ltd. (Shaoxing, China).

### 2.2. Mix Proportions and Mechanical Properties of the CA Mortar

**Figure 1:**
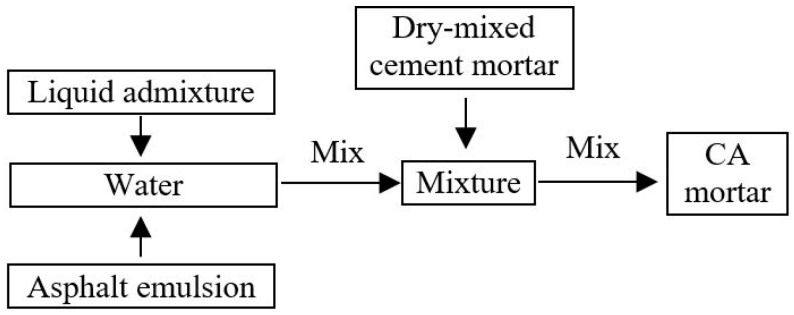
Preparation of the CA mortar.

The mix proportions of the CA mortar used for the fatigue test is shown in Table 3 and the mixing method is illustrated in Figure 1. After mixing, the properties of the fresh CA mortar were tested and was found to meet the requirements of the provisional technical conditions in China. The cylindric CA mortar specimen with Φ 50 mm × 50 mm were prepared and cured under the standard curing condition. The compressive strength and modulus of elasticity standard curing after 1 d, 7 d and 28 d were detected and the results are shown in Table 4. As seen in Table 4, the mechanical properties of the CA mortar can meet the requirements of the interim technical conditions.

### 2.3. Fatigue Testing

#### 2.3.1. Fatigue Testing Device

**Figure 2:**
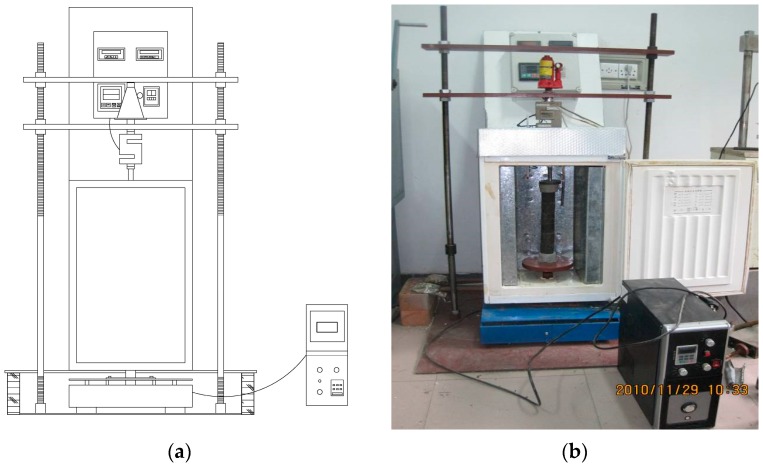
The setup developed for the fatigue testing of the CA mortar. (**a**) diagrammatic sketch of the setup and (**b**) the samples laid in the setup.

In this study, an anti-fatigue experimental device for fatigue testing of the CA mortar was developed in order to simulate the actual serving condition, including the load, the high-frequency vibration, the freeze-thaw cycle, the high-low temperature cycles, etc., as shown in Figure 2. By using the setup, the stress loaded, the environmental temperature and the vibration frequency can be controlled through computer-programming for digital signal. The high-frequency vibration is simulated by vertical vibration motor. The system is suitable for dynamic testing and the loading mode is the stress control. The initial static load stress is fixed and the electromagnetic vertical vibration machine is used to produce vibration. All loading modes can be controlled by closed-loop feedback circuit except the dynamic loading. The automatic control system has two control channels, vertical vibration loading and temperature change independently and synchronously. The device can simulate the actual service environment of the CA mortar in the ballastless slab track. During the fatigue test, the specimens were installed as shown in Figure 2b.

#### 2.3.2. Temperature Range

**Figure 3:**
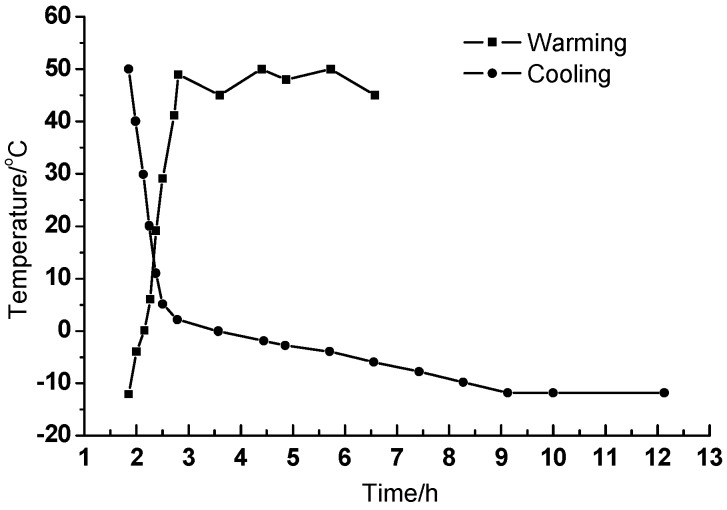
The temperature curve during fatigue testing.

Take Beijing as a typical area to decide the temperature range during the fatigue test. Considering in summer the temperature of the CA mortar under the slab may reach over 50 °C under the sunshine and in winter the temperature may decline to about −12 °C in Beijing, the temperature range were set between −12 °C and 50 °C. As an initial experiment in the lab, because the cooling rate is a little slow but the heating rate is relatively rapid, the cooling time was set for 12 h, and the heating time was set as 5 h, and finally the temperature can be controlled at 50 °C and −12 °C for 4 h respectively. The final temperature curve is shown in Figure 3 and the total fatigue testing time is set at 180 h.

#### 2.3.3. Vibration Control

In ballastless slab track, when the train passes at a high speed, the load on track structure can be generally divided into vertical load, horizontal load and vibration load, and the vibration of the CA mortar in track is mainly vertical vibration [24]. The train passes through and produces a vibration wave. The amplitude and frequency of the vibration wave are variable. Therefore, an electromagnetic vertical vibration test machine is selected in this study.

The vibration simulation of the device is controlled by an electromagnetic vibration tester, and its frequency control range is 1–400 Hz. A great deal of theoretical and experimental work done by the British Railway Technology Center over the years has shown that the main causes of vertical wheel-rail force are resulted from the irregularities and the local flat scars around the wheel [28,29,30]. The experiments showed that the vertical wheel-rail force mainly occurs in 3 frequency ranges: (i) The low frequency range (0.5~10 Hz), which is almost entirely caused by the relative movement of the vehicle body to the suspension part; (ii) intermediate frequency range (30~60 Hz) due to the springback effect of spring wheel on mass; and (iii) the high frequency range (100–400 Hz) which is caused by the resistance of rail to the wheel rail contact surface. The measured results show that the wheel-rail force is more intense in the middle frequency range, and the high frequency range mainly affects the dynamic response of the car body [28]. Considering that the vertical load of train vibration load is much larger than the horizontal load, the vertical wave was mainly considered in the study.

For asphalt pavements [31,32,33], the loading time of wheel loads in laboratory tests can be determined by Van der Poel’s Formulas (1) and (2):

(1) t=12Ωf

When the loading frequency is 20 Hz, the corresponding loading time is:

(2) t=12Ωf=0.032s

The loading time of 0.032 s is approximately corresponding to the 120 to 130 km/h for the vehicle running on the asphalt mixture pavement surface. Assume that the wheel of a train is similar to the wheel of a car, the loading frequencies on the CA mortar corresponding to the train speed of 200 km/h and 350 km/h can be calculated and to be about 33 Hz and 58 Hz, respectively. As the speed of the train increases, the vibration shifts to a high frequency. According to Li Shiyi [34], when the train runs at the speed of 200, 250 and 300 km/h on Qin-Shen high-speed railway, the sleeper frequency increases from 93.3 Hz to 116.7 Hz and 140.0 Hz according to the spectrum curve of ground vibration acceleration at 8.0 m track.

Based on the comparison between theoretical calculation and the actual measurement in Qinhuangdao-Shenyang high-speed railway in China, the frequency range of the CA mortar in Ballastless slab track is in the range of 0–100 Hz. During the fatigue test, the vibration frequencies are set to 0, 25 Hz, 50 Hz, 75 Hz and 100 Hz respectively.

#### 2.3.4. Load Control

According to the maximum stress of the CA mortar layer resulted from the axle load of the CRH2 high-speed train to be around 49.63 kPa [35], the load on the CA mortar specimen should be 0~4000 N, and the stress should be 0~2.0 MPa correspondingly. Therefore, in this study, the static load is set to 0.5 kN, correspondingly the compressive stress is about 0.25 MPa. During the test, it was found that the dynamic load of the specimen is between 2.0 kN and 2.5 kN at different frequencies.

### 2.4. Mechanical Properties

After the fatigue testing, the CA mortar specimen was placed in the standard curing box for 24 h. Then the load-displacement curve of the CA mortar specimens with and without polymer emulsion incorporation was determined at the same age as the fatigue test specimens, and the stress-strain curve of the CA mortar under different test conditions was obtained through data processing, and the peak stress *σ_p_* was obtained. At the same time, the elastic modulus of the CA mortar was calculated, which is represented by the secant modulus, that is, the slope of the secant line corresponding to the 0.3*σ_p_*–0.5*σ_p_* in the curve.

### 2.5. Microstructure

Hitachi S-4700 scanning electron microscope (Hitachi, Tokyo, Japan) was used to observe the microstructure of the CA mortar samples before and after the fatigue test, and Autopore VI 9500 mercury intrusion porosimetry (MIP) was used to analyze the pore size distribution of CA mortar before and after the fatigue test.

## 3. Results and Discussion

### 3.1. Stress-Strain Curves of the CA Mortar before and after Fatigue Test

**Figure 4:**
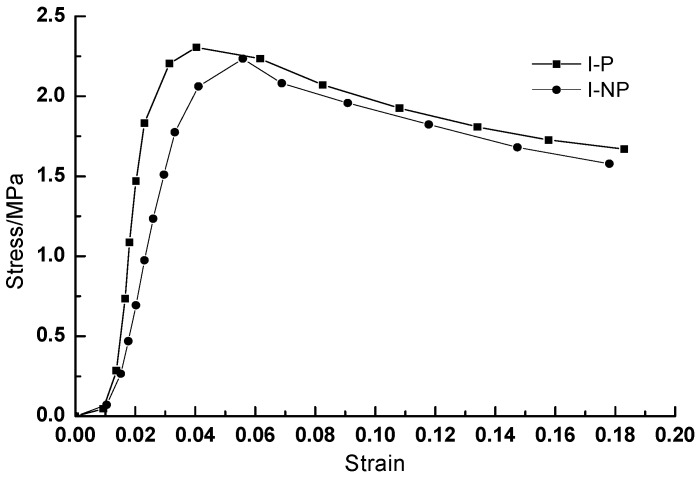
Stress-strain curve of the CA mortar after standard curing for 28 days.

Figure 4 shows the stress-strain curves of the CA mortar with and without polymer emulsion after standard curing for 28 d. As seen in Figure 4, the initial slope of the stress-strain curve of the CA mortar with and without polymer emulsion is relatively small, mainly because the two ends of the specimen are not completely flat, so the stress grows slowly along with the axial strain. When the axial compression deformation exceeds the uneven height, the stress and axial strain grow linearly. However, after the peak load of the CA mortar, the decrease is relatively slow. The interpenetrating network structure formed by asphalt and cement has significantly improved the fracture toughness of mortar, making it not drop rapidly after the peak stress as ordinary mortar, which indicates that the CA mortar possess better toughness and ductility. At the same time, it can be found that the strength and elastic modulus of the CA mortar with polymer emulsion are both higher than that without polymer emulsion under the same conditions, as indicated in Figure 4.

**Figure 5:**
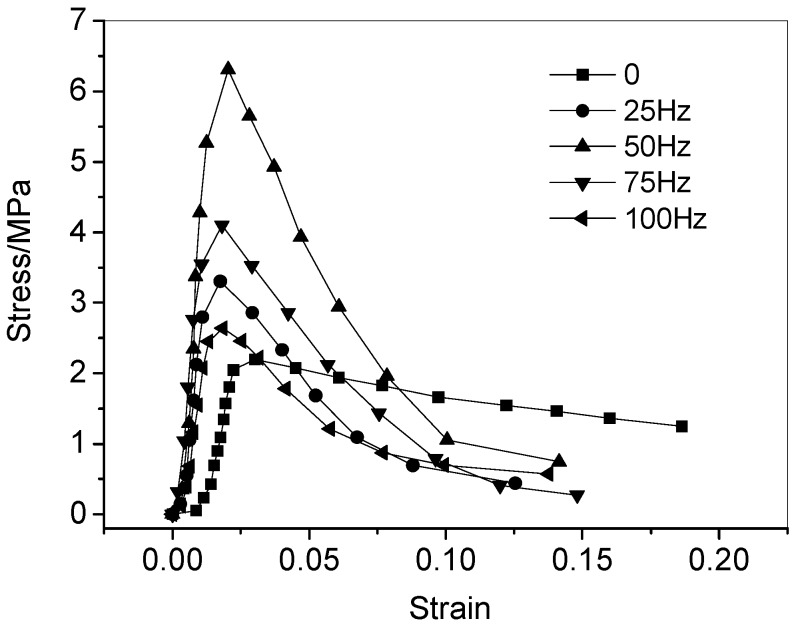
Stress-strain curves of the CA mortar with polymer emulsion incorporated after fatigue test under various frequencies.

**Figure 6:**
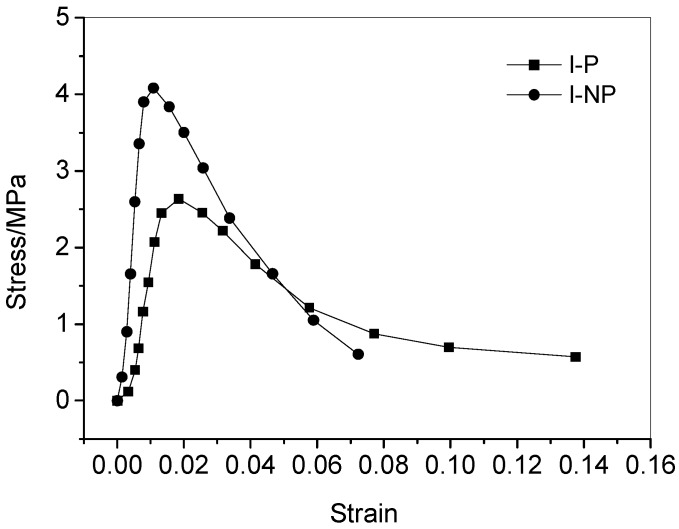
Effect of polymer emulsion incorporation on the stress-strain curve of the CA mortar after fatigue test (100 Hz).

One of the mechanical properties of the viscoelastic materials is that they are sensitive to the strain rate, which is related to the response characteristics of the strain rate of the purely viscous damper unit that constitutes its structure. Therefore, the characteristics of the purely viscous damper unit in simulated service at different vibration frequencies will change, which is manifested as the change of the stress-strain state of materials after service. Figure 5 shows the full stress-strain curves of the CA mortar with polymer emulsion after fatigue test at different vibration frequencies. Figure 6 illustrates the full stress-strain curves of the CA mortar with and without polymer emulsion after fatigue test at the vibration frequency of 100 Hz. Table 5 presents the mechanical properties of the CA mortar after fatigue test.

The full stress-strain curve of the material under pressure reflects the compressive performance of the material. It can be seen from Figure 5 that the initial stage of the stress-strain curve for the CA mortar after fatigue test is similar to that of the CA mortar under the standard curing condition, both of which are linear. With the increase of the axial pressure, nonlinear deformation occurs in the CA mortar, and stress-strain curve bends after reaching the peak value. As compared with the CA mortar under the standard curing conditions, the curve drops rapidly, and the deformation capacity is poor, representing a typical brittle material characteristic. For the test without vibration load, that is, only suffering with the freeze-thaw cycle, the strength and elastic modulus of the CA mortar after fatigue test are reduced to a certain extent as compared to that under the standard curing situation. The reason may be that freezing of the free water inside the CA mortar may generate a certain expansion stress and cause the internal pores larger, so the mechanical properties of the CA mortar after the test decreased. However, for the test with vibration during the fatigue test, the strength of CA mortar increased from 2.600 MPa to 6.306 MPa after fatigue within the range of medium and low frequencies (≤50 Hz), and the elastic modulus increased from 160.0 MPa to 868.9 MPa after fatigue test as compared to that under the standard curing at the same curing age. In the range of high frequency (>50 Hz), the strength and elastic modulus of the CA mortar after the fatigue test still increased to a certain extent, as compared to that under the standard curing at the same curing age, but less than those while testing at the low frequency and at the same curing age. During the fatigue test, if the CA mortar is subjected to both high frequency vibration and high temperature, the excess free water in the sample will leak out under high frequency vibration. Meanwhile, the asphalt in the hardened CA mortar may soften first, then migrate and fill into the large pores, thus the mortar may become denser and both the compressive strength and the elastic modulus will be enhanced. Furthermore, under the heat & cold cycles and repeated continuous loading, the asphalt in the hardened CA mortar may also gradually become harder and harder [36]. When subjected to both high frequency vibration and low temperature, the free water inside the pores could not crystallize. When the vibration frequency is too high, typically the more the fatigue loading times of the material are, and the more accelerated damage accumulation inside it is, thus the strength of the CA mortar in the high frequency range does not increase too much as compared to that tested under the lower frequency, and may even be lower than that of the mortar under the standard curing.

**Figure 7:**
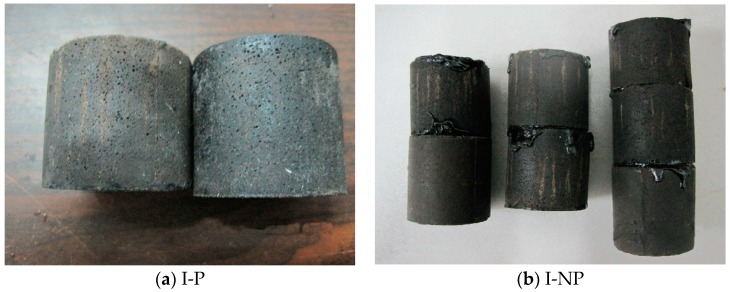
Effect of the polymer emulsion addition on the oozing of the asphalt in the hardened CA mortar during the fatigue test. (**a**) the CA mortar with polymer emulsion incorporation and (**b**) the CA mortar without polymer emulsion.

From Figure 6 and Table 5, it can be seen that the addition of polymer emulsion can significantly improve the anti-fatigue performance of the CA mortar. Under the same fatigue test conditions, the increase of elastic modulus is significantly reduced. The elastic modulus of the CA mortar without polymer emulsion is almost three times that of the one with polymer emulsion after the fatigue tests. The gradually increase of the elastic modulus makes the CA mortar gradually lose its function as an elastic adjustment layer. The stress-strain curve drops rapidly behaving like the normal cement mortar. This may be due to the fact that the asphalt in the CA mortar without polymer emulsion addition is prone to ooze out during the fatigue test process, whereas the oozing phenomenon did not happen for the mortar with polymer emulsion addition, as revealed in Figure 7.

### 3.2. Microstructure Analysis

**Figure 8:**
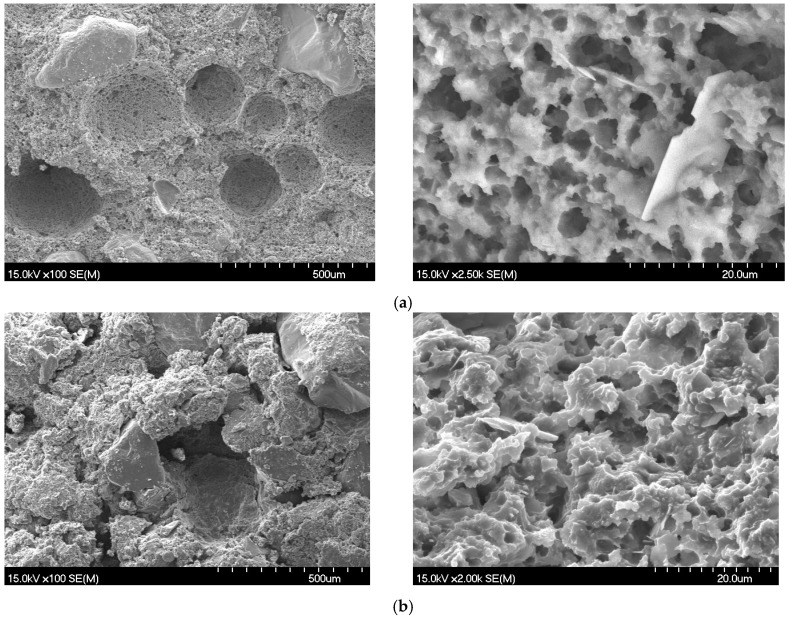
SEM photograph of the CA mortar (**a**) under the standard curing condition and (**b**) after the fatigue test at 50 Hz but at the same total curing age.

In order to reveal the reason why the strength and elastic modulus increase but the toughness and ductility of the CA mortar decrease after the fatigue test under the high-frequency vibration, together with the load and the freeze-thaw cycles, the changes of the microstructure and the pore size distribution of the hardened CA mortar were detected through the SEM observation and MIP test for the samples with and without suffering the fatigue test, but at the same curing age. Figure 8 illustrates the SEM photograph of the CA mortar after the fatigue test at 50 Hz as compared to that at the standard curing and at the same curing age. As can be seen from Figure 8, the asphalt and cement in the CA mortar under the standard curing form an interpenetrating network structure with large porosity. The macroscopic large pores showed spherical, which are typically bubbles resulted from the air-entraining agent during mixing the CA mortar. The microscopic pores are uniformly distributed and the pore wall is not damaged. After the fatigue test, the internal pore structure of CA mortar is significantly affected by the fatigue of the load and the vibration. The internal interpenetrating network structure is obviously deformed, among which the large pore is obviously damaged by vibration and the pore wall is seriously deformed. Although no obvious damage was found in the pore wall of the microstructure pore, the micropore porosity decreased significantly and the structure became more compact, which was manifested as the strength and elastic modulus increased, the ductility and toughness decreased, after the fatigue test. At the same time, the flowing along the internal pores and the filling of the softened asphalt into the large pores under the high frequency vibration and high temperature can also be observed, as seen in Figure 8d.

**Figure 9:**
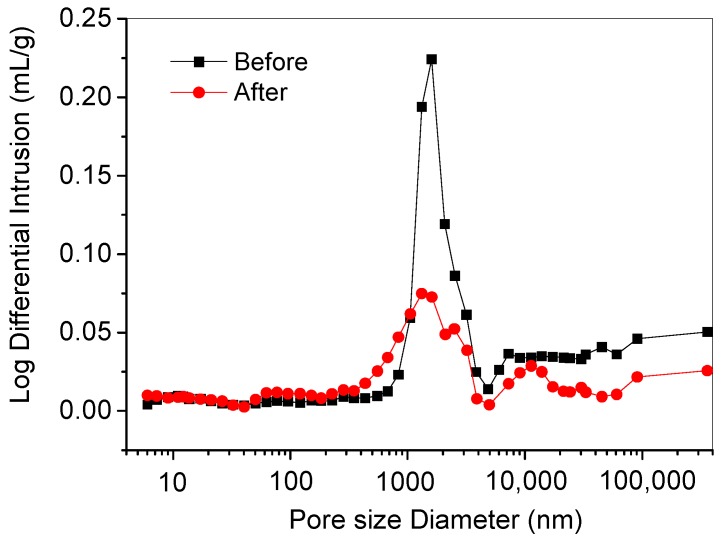
Pore size distribution of the CA mortar under the standard curing condition and after the fatigue test at 50 Hz but at the same total curing age.

Figure 9 and Table 6 present the pore size distribution and the characteristic parameters of the pore structure for the CA mortar under the standard curing condition and after the fatigue test at 50 Hz but at the same curing age. As can be seen from Figure 9, the pore volume of the CA mortar greater than 1000 nm increased significantly after the fatigue test at 50 Hz, while the pore volume within the range of 50–1000 nm increased slightly, and the pore volume of less than 50 nm remained basically unchanged. Typically, this is caused by the migration of the softened asphalt to fill the pores in the CA mortar under the coupling effect of the vibration, load and high temperature, thus improve the density of the CA mortar significantly. From Table 6, it can also be seen that both the average pore volume and the average pore diameter of CA mortar after fatigue test are significantly smaller than that cured under the standard curing condition at the same curing age.

### 3.3. Effect of Vibration Frequency on the Properties of the CA Mortar after the Fatigue Test

**Figure 10:**
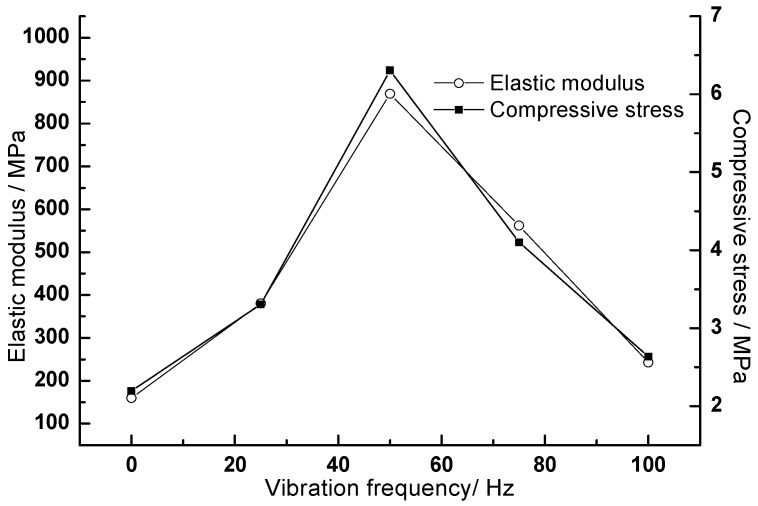
Effect of the vibration frequency on the properties of the CA mortar after the fatigue test.

Figure 10 presents the effect of vibration frequency on the compressive strength and elastic modulus of the CA mortar after the fatigue test. As seen in Figure 10, the compressive strength and the elastic modulus of the CA mortar showed an increase firstly then a decrease with increasing the vibration frequency during the fatigue test, and at the frequency of 50 Hz, a maximum compressive strength and the elastic modulus is obtained. The reason is not clear, which may be due to that the natural frequency of the CA mortar in this study is about 50 Hz, the resonance occurred between the CA mortar and the equipment under this frequency, thus showed the greatest influence on the properties of CA mortar, which needs further research.

According to the effect of the vibration frequency on the properties of the CA mortar, it seems that the CA mortar prepared for the CRTS I ballastless slab track should be carefully designed and controlled in advance in order to avoid the resonance and prevent the fatigue failure of the CA mortar, thus be able to ensure the elastic adjustment function of the CA mortar and the running safety of the track.

## 4. Conclusions

The CA mortar used in the ballastless slab track may encounter the coupling effect of the high-frequency vibration, load, and high-and-low temperature cycles during service, and the deterioration under fatigue may happen during service of the high-speed railway. In this study, the performance degradation and its mechanism of the CA mortar with and without polymer emulsion incorporated under the coupling fatigue effects of the high-frequency vibration, load and temperature were studied by using an anti-fatigue testing device specially developed for the CA mortar used in the ballastless slab track of the high-speed railway. The conclusions can be obtained as follows:

(1) The deformation capacity of the CA mortar for CRTS I slab ballastless slab track decreased after fatigue test under simulated service environment, presenting a typical brittle characteristic and an obvious reduction of the ductility and toughness. The variation of compressive strength and elastic modulus of the CA mortar after the fatigue test is approximately in a piecewise linear relationship with the increase of vibration frequency. The strength and elastic modulus are reduced under freeze-thaw action only without vibration as compared to that cured at the standard curing condition.

(2) The SEM observation and the MIP analysis showed that the internal pore structure of the material changed after the fatigue test. The volume of the macropore decreased whereas that of the micropore increased. Meanwhile, the asphalt in the hardened CA mortar revealed a softening and migration from the bulk paste to fill the pore and make the structure denser and even ooze out of the CA mortar under the high-frequency vibration and high temperature.

(3) Through incorporating the polymer emulsion, the anti-fatigue property of the CA mortar was obviously improved, which can prevent the CA mortar from losing its elastic adjustment function too early. The performance of CA mortar without polymer emulsion is more similar to that of ordinary mortar after the fatigue test. Though increase of the strength and elastic modulus for the CA mortar after severe service is beneficial to the stability of train running, the comfort level and safety of the train operation may decline due to the gradual reduction of the ductility & toughness and the gradual loss of the elastic damping adjustment function of the CA mortar layer between the base concrete slab and the track slab.

## Figures and Tables

**Table 1:** Properties of the asphalt emulsion.

Engler’s viscosity (25 °C)/s		7.8
Particle polarity		Positive
Mixing stability with cement/%		0.1
Residue on sieve (1.18 mm)/%		0.00
Storage stability (1 d, 25 °C)/%		0.1
Storage stability (5 d, 25 °C)/%		2.3
Storage stability at −5 °C		Qualified
Evaporation residue	Content of residue/%	62.5
	Penetration (25 °C, 100 g)/0.1 mm	85.0
	Ductility (15 °C)/cm	118
	Solubility (trichloroethylene)/%	99.2

**Table 2:** Properties of the dry-mixed cement mortar for the CA mortar.

Expansion Ratio/%	Compressive Strength/MPa		
	1 d	7 d	28 d
2.52	11.7	35.9	60.2

**Table 3:** Mix proportion of the CA mortar used in CRTS I ballastless slab track.

Serial Number	Asphalt Emulsion/mL	Dry-Mixed Mortar/g	Water/mL	Polymer Emulsion/mL	De-Foaming Agent/mL
I-P	442	1000	50	50	1
I-NP	442	1000	80	0	1

**Table 4:** Mechanical properties of the hardened CA mortar used for the fatigue test.

Serial Number	Compressive Strength/MPa			Elastic Modulus/MPa
	1 d	7 d	28 d	28 d
I-P	0.26	1.2	2.6	160
I-NP	0.13	1.0	2.0	130

**Table 5:** Properties of the CA mortar after fatigue test under various vibrating frequencies.

CA Mortar	Frequency (Hz)	Stress Peak (MPa)	Strain at Peak Stress (%)	Elastic Modulus (MPa)
I-P	Standard curing	2.310	4.036	160.0
I-NP		2.234	5.586	130.0
I-P	0	2.194	3.027	159.8
	25	3.306	1.755	380.1
	50	6.306	2.048	868.9
	75	4.101	1.821	561.6
	100	2.636	1.838	242.6
I-NP	100	4.083	1.087	745.7

**Table 6:** Characteristic parameters of the pore structure for the hardened CA mortar before and after fatigue test.

Serial Number	Total Intrusion Volume (mL/g)	Median Pore Diameter (Volume) (nm)	Median Pore Diameter (Area) (nm)	Average Pore Diameter (4 V/A) (nm)
After fatigue test	0.0971	1853.3	11.6	153.4
Standard curing	0.1641	2862.8	11.8	299.5
